# Stratification of Gene Coexpression Patterns and GO Function Mining for a RNA-Seq Data Series

**DOI:** 10.1155/2014/969768

**Published:** 2014-05-19

**Authors:** Hui Zhao, Fenglin Cao, Yonghui Gong, Huafeng Xu, Yiping Fei, Longyue Wu, Xiangmei Ye, Dongguang Yang, Xiuhua Liu, Xia Li, Jin Zhou

**Affiliations:** ^1^Department of Hematology, The First Affiliated Hospital, Harbin Medical University, Harbin 150001, China; ^2^Health Ministry Key Lab of Cell Transplantation, Harbin 150001, China; ^3^Heilongjiang Institute of Hematology and Oncology, Harbin 150001, China; ^4^College of Bioinformatics Science and Technology, Harbin Medical University, Harbin 150081, China; ^5^College of Life Science, Heilongjiang University, Harbin 150080, China

## Abstract

RNA-Seq is emerging as an increasingly important tool in biological research, and it provides the most direct evidence of the relationship between the physiological state and molecular changes in cells. A large amount of RNA-Seq data across diverse experimental conditions have been generated and deposited in public databases. However, most developed approaches for coexpression analyses focus on the coexpression pattern mining of the transcriptome, thereby ignoring the magnitude of gene differences in one pattern. Furthermore, the functional relationships of genes in one pattern, and notably among patterns, were not always recognized. In this study, we developed an integrated strategy to identify differential coexpression patterns of genes and probed the functional mechanisms of the modules. Two real datasets were used to validate the method and allow comparisons with other methods. One of the datasets was selected to illustrate the flow of a typical analysis. In summary, we present an approach to robustly detect coexpression patterns in transcriptomes and to stratify patterns according to their relative differences. Furthermore, a global relationship between patterns and biological functions was constructed. In addition, a freely accessible web toolkit “coexpression pattern mining and GO functional analysis” (COGO) was developed.

## 1. Introduction

High-throughput RNA sequencing (RNA-Seq) is a revolutionary technology in the postgenome era. RNA-Seq rapidly generates transcript sequences and provides more detailed information than microarray-based technologies. RNA-Seq has the ability to reconstruct a complete map of the transcriptome in different cell types or physiological conditions [1, 2]. The dynamic transcriptome of cells is an important molecular signature that can represent the physiological state of different tissues, facilitating an understanding of the mechanism of gene regulation. RNA-Seq technology is becoming increasingly common as the sequencing cost is reduced and the accuracy is improved. More studies use RNA-seq technology, resulting in a series of RNA-Seq datasets across multiple related experimental conditions, such as in comparisons of multiple tumor subtypes or the effect of the concentration of a drug. Genes that exhibit similar responses to external stimuli are potentially controlled by similar regulatory mechanisms [3]. Therefore, it is important to monitor the expression pattern of genes and to discover the genes that are coexpressed among multiple conditions. These coexpression patters could describe the biological regulatory relationships of these genes.

Since the emergence of RNA-Seq technology, many differential expression (DE) analysis methods based on RNA-Seq data have been developed, such as Cuffdiff [4], DESeq [5], edgeR [6], and SAMseq [7]. These methods have been extensively used for differential expression analysis between two conditions. Numerous genes related to specific biological functions have been found by these bioinformatics methods and confirmed by follow-up biological experiments [8, 9]. However, the DE methods described above were developed for pairwise comparisons, creating cumbersome, and confusing analyses when processing data from more than two conditions. In addition, a functional analysis was performed for only the DE genes that were isolated from the whole transcriptome, overlooking useful additional gene expression information. Because of the gene dosage effect, genes that are only slightly differently expressed may still provide useful information as a measure of functional status [10, 11]. Even the overlooked stably-expressed genes may be more essential for the survival of an organism [12].

**Figure 1:**
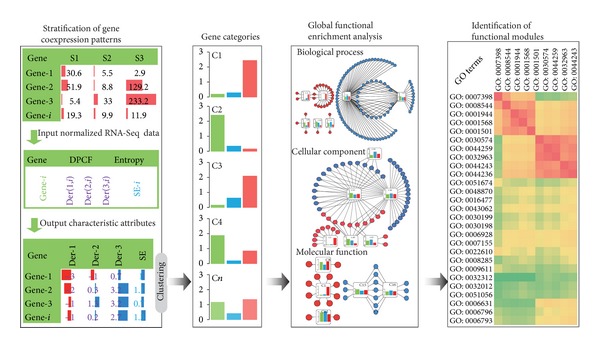
A schematic overview of COGO. A series of RNA-Seq data with three conditions was selected to illustrate the analysis process. The characteristic attributes “Der” and “SE” were extracted by a derivation method of polynomial curve fitting (DPCF) and by Shannon's Entropy (SE) models, respectively. Gene categories can then be established through clustering. A functional enrichment analysis was then performed for the categories to determine significant functions. Finally, the semantic similarity measurement was conducted to identify functional modules.

Therefore, we developed an integrated strategy for differential coexpression pattern and GO function mining (COGO) for a RNA-Seq data series. The COGO strategy enables the biologist to view the data from a global perspective (Figure 1). First, the characteristic attributes should be extracted from the expression values of a series of RNA-Seq datasets. Second, the expression patterns can be established and stratified according to feature attributes that were extracted. Finally, functional enrichment analyses are performed for each category to determine significant function terms and the functional relationships of different GO terms that are obtained by measuring their functional semantic similarity [13]. The algorithms used in COGO are detailed in Section 2 and in Figure 1.

To illustrate a typical analysis, we applied a published RNA-Seq dataset obtained from the Gene Expression Omnibus (GEO) that contains three biological conditions [14]. The results indicated that genes coexpressed in specific categories could represent the response and stability of biological functions to the experimental conditions. In addition, a web toolkit, “COGO”, was developed based on this method (http://202.97.205.74:8080/COGO). Users of this toolkit submit a profile of RNA-Seq data and receive stratified gene coexpression categories and the affected functional modules.

## 2. Methods

### 2.1. Differences in Gene Expression among Multiple Groups

Gene expression levels were quantified and normalized as FPKM/RPKM measurements. The Cufflinks package was used to calculate gene expression values using default settings [15]. Then, the average gene expression level was calculated for the experimental replicates. To identify coexpression patterns of a series of RNA-Seq libraries with *M*(*M* ≥ 3) experimental conditions in one study, we first quantified gene expression differences among multiple conditions. We defined *e*_*i*,*j*_ as the expression value of gene *i* = {1,…, *N*} of condition *j* = {1,…, *M*}, where *N* is the number of genes in the dataset. We adopted a method that was based on Shannon's Entropy (SE). SE has been used previously to identify DE genes and alternative splicing in gene expression data [16]. In this procedure, SE was introduced to measure the differences in gene expression values across experimental conditions and was defined as follows:

(1) $$\begin{array}{c} \text{S}{\text{E}}_{i}=-\overset{M}{\underset{j=1}{\sum }}\frac{ae_{i,j}}{S_{i}}\text{lo}{\text{g}}_{2}\left(\frac{ae_{i,j}}{S_{i}}\right). \end{array}$$

A tiny value *α* was added to the expression value *e*_*i*,*j*_ to avoid 0 values. The new expression value was *ae*_*i*,*j*_ = *e*_*i*,*j*_ + *α*, and the sum of the expression value of gene *i* among *M* experimental conditions was calculated as *S*_*i*_ = ∑_*j*=1_^*M*^ *ae*_*i*,*j*_.

### 2.2. Attributes Extraction according to Gene Expression Trends

SE could measure differences in variable elements, but was unable to determine the specific expression patterns within a calculation unit. Therefore, we introduced a pattern mining method based on a derivation method of polynomial curve fitting (DPCF) to describe the expression patterns of a specific gene among multiple conditions [17]. To facilitate the pattern mining of genes, the gene expression values were normalized because the polynomial fitting coefficients and fitted values are positively correlated. We defined a new dimensionless expression value, *en*_*i*,*j*_ = *ae*_*i*,*j*_ *M*/*S*_*i*_, as the gene relative-expression level among multiple conditions. Then, the polynomial fitting formula was defined as *y* = *f*_*i*_(*x*), *x* ∈ (1 ⋯ *j* ⋯ *M*). The derivative is a measure of how a function changes and the response of the curve trend as the inputs change. Therefore, the derivative function value of each experimental point was obtained from the following clustering attribute formula:

(2) $$\begin{array}{c} \text{De}{\text{r}}_{i}=f_{i}^{\prime }\left(x\right),\;x\in \left(1\cdots j\cdots M\right). \end{array}$$

The changes in the gene expression trend between successions of conditions could be represented by Der_*i*_. The arrangements of data should influence the discovery of the effect of expression patterns. Therefore, the order of the data must be consistent with the properties of the study, for example, sorting data according to a drug concentration gradient or tumor stages of development.

### 2.3. Clustering to Mine Coexpression Patterns

The determination of DE genes was obscured by the fact that a 2-fold-change may not be more meaningful than a 1.5-fold-change at the level of biological function. Therefore, we aimed to discover the expression patterns that led to different phenotypes. A hierarchical clustering method was applied, which sought to create a hierarchy of clusters in an unsupervised classifier [18]. To decide which genes should be combined in a cluster, a measure of dissimilarity in the sets of attributes was obtained. A distance matrix was constructed with *M* + 1 attributes and *N* genes, and then the hierarchical clusters were built by progressively merging clusters. To construct a relatively objective map of the transcriptome, the default value for the cluster number (CN) was defined as follows:

(3) $$\begin{array}{c} \text{CN}=\left\lfloor \left(M+1\right)\right\rfloor \text{lo}{\text{g}}_{10}N. \end{array}$$

However, we zoomed in/out of the map by changing the value of CN(1 < CN < *N*) if rigorous expression patterns were needed for detailed analysis. Then, categories of coexpression genes were obtained and represented as *Cn*  (1 < *n* < CN), and the gene number of category *Cn* is *N*_*n*_. The gene expression patterns of categories were represented by the average of the gene relative-expression level, which is defined as *A* *en*_*Cn*,*j*_ = ∑_1_^*N*_*n*_^ *en*_*i*,*j*_/*N*_*n*_, *j* ∈ (1 ⋯ *M*). Therefore, stably expressed and unstably expressed categories among multiple conditions were divided by the following criteria:

(4) $$\begin{array}{c} \frac{\max\left(Aen_{Cn,j}\right)}{\min\left(Aen_{Cn,j}\right)}\{\begin{array}{cc} \begin{array}{c} \leq \beta ,\text{Stable}\;\;\text{expressed},\\ >\beta ,\text{Unstable}\;\;\text{expressed}, \end{array} & j\in \left(1\cdots M\right), \end{array} \end{array}$$

where *β* was defined as the Relative Average Expression Difference (RAED) and was set to 1.2 as default, which is more stringent than the fold-change cutoff value of “2” and can be defined by users [19].

### 2.4. Global Functional Enrichment Analysis

To explore the biological relationships of genes in the categories obtained by our method, a functional enrichment analysis (FEA) was introduced for the gene categories using DAVID [20]. The goal of the enrichment analysis was to determine which biological functions might be predominantly affected in the set of genes with identical expression patterns among different experimental conditions [21]. We established the Gene Ontology categories as the background knowledgebase of the FEA to acquire the functional annotating concepts for each gene category and arrive at a profile of the biological function or mechanisms. Performing a FEA on all categories was meaningful because we were able to explore the effect of external factors or physiological state on the stability of gene expression or on the biological function. To elucidate the mechanisms of regulation, a semantic similarity measurement in GO terms was conducted to identify functional modules [13].

## 3. Application and Results

### 3.1. Data Acquisition

To examine the newly developed expression pattern classifying method, published RNA-Seq data were obtained from the GEO (http://www.ncbi.nlm.nih.gov/geo/, accession number GSE33782). The data contains three RNA-Seq libraries from a colorectal cancer patient: cancer (C), paracancer (P), and distant normal tissues (N). To avoid potential biases, the datasets were filtered according to the status code provided by the Cufflinks and the FPKM value; all expression levels for a specific gene among samples were reliable (status code is OK), and the average of the gene's FPKM among samples was greater than 2. In total, 11,969 genes were detected as expressed in at least one of the samples (see Table S1).

### 3.2. Coexpression Pattern Mining

**Figure 2:**
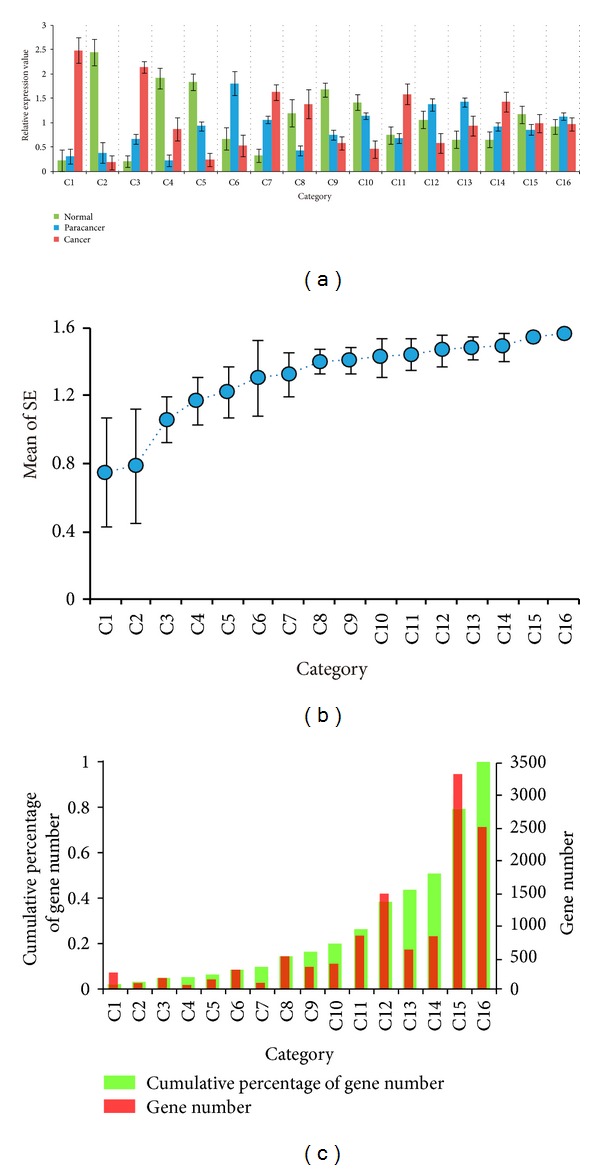
Gene expression pattern classification results of the colon RNA-Seq dataset. (a) A chart showing gene expression patterns among different tissues for each cluster category. The *y*-axis is dimensionless and represents the mean gene relative expression level; error bars show the standard deviation. (b) The hollow dots represent the mean of SE for each category; error bars show the standard deviation. (c) The number of genes in each category and the cumulative percentage of the number of genes from C1 to C16.

The characteristic attributes were computed and genes were clustered into 16 categories using the defined formula (see Section 2). The results showed that the genes with similar expression patterns among different types of tissues clustered into identical categories (Figure S1(a)). For example, transcripts in C1 were absent in or at a very low level in normal tissues and paracancer tissues; however, these transcripts were expressed at relatively high levels in cancer tissues (Figure 2(a)). Similarly, genes in C2 were expressed at low levels in paracancer tissues and cancer tissues, but were expressed at high levels in normal tissues. In general, the gene expression differences among the three types of tissues gradually reduced from C1 to C16 (Figure 2(a)). We conducted a statistical analysis of the number of gene and average entropy of each category and then calculated the category frequency over the total number of genes. The mean of the SE of the categories gradually increased, which represents a decrease in the expression difference treads from C1 to C16 (Figure 2(b)). The majority of the genes were gathered in higher-numbered categories, which was in agreement with real biological situations (Figure 2(c)) [22]. The gene expression differences of the categories were determined using a stringent default value. The results showed that the top 14 categories accounted for 51.2% of the total genes and had differences in various degrees, and 48.8% of genes in the last two categories were stably expressed. These results indicated that the expression levels of most genes were relatively stable among different physiological states; this finding is consistent with the assumption that most genes are equivalently expressed at different conditions [22, 23] (Figure 2(c)). Furthermore, the number of significant upregulated genes exceeded the downregulated genes. The overrepresentation of upregulated gene transcripts is likely because of the metabolic exuberant state of cancer cells promoting related genes to be upregulated. Therefore, upregulated genes may be more involved in the process of tumor formation compared to downregulated genes (Figures 2(a) and 2(c)).

### 3.3. Functional Enrichment Analysis

**Figure 3:**
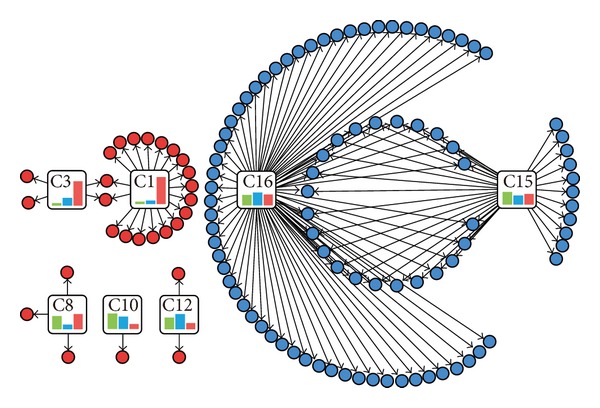
The functional relationship network of categories and enriched GO terms for the biological process category. The enriched GO terms of C15 and C16 are indicated by blue circles, and the other categories are indicated by red circles. The bar charts represent the expression pattern of the category. This figure was constructed to show the overall relationship of GO functions to gene patterns and gene patterns to gene patterns. More detailed GO terms are presented in Table S2 in Supplementary Material available online at http://dx.doi.org/10.1155/2014/969768.

**Figure 4:**
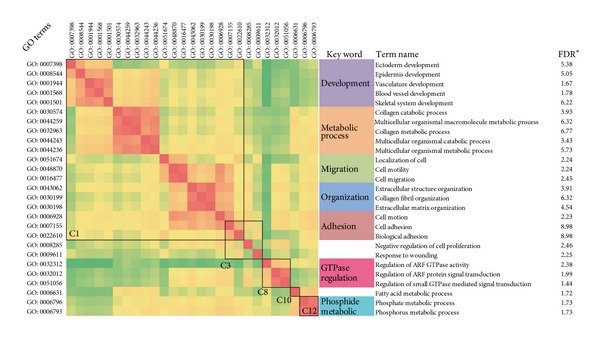
The functional similarity of the GO terms in the biological process category enriched in C1, C3, C8, C10, and C12 are displayed as a heatmap, and the similarity scores are indicated by color intensity, with red representing high similarity and green representing low similarity (FDR* = −log_10_(FDR)).

Most cancers, including colon cancer, are complex and can be caused by multiple genes and interactions. With the advance of high throughput technologies, it is now feasible to reverse engineer the underlying genetic networks that describe the interplay of molecular elements that lead to complex diseases. To explore the biological relationship of coexpressed genes obtained by our method, a FEA was performed for the gene categories using DAVID [20]. The gene ontology (GO) analysis revealed that not every category was significantly enriched for GO terms, but the number of GO terms that were significantly enriched in C15 and C16 substantially exceeded the other categories (Figure 3, Figure S2, and Figure S3). This finding suggested that the majority of the core physiological function of the cell remains stable, such as “cell death” and the “cell cycle.” The FEA identified 23% of the significantly enriched terms in the biological process category to be associated with dysfunctional terms (see Table S2). However, the percent of dysfunctional terms (23%) is not proportional to the percent of differentially coexpressed genes (51.2%). This indicated that the abnormality of colon cell proliferation is because of the abnormal expression of related genes, but there were differentially expressed genes independent of experimental factors. To elucidate the mechanism of gene regulation, a functional relationship in enriched GO terms can be discovered by measuring their functional semantic similarity. Although we chose a relatively lenient cluster number by default, we still discovered enriched GO terms consistent with previous studies, such as “ectoderm development,” “collagen catabolic process,” and “cell migration” [24, 25]. Some functional modules were identified for specific categories, such as functions related to “development,” “metabolic process,” and “migration” (Figure 4). For example, the significant GO terms in the biological process category in C1 can be classified into 5 functional modules by the GO semantic similarity method and summarized by keywords; the “development” subtype, including “ectoderm development,” “epidermis development,” “vasculature development,” “blood vessel development,” and “skeletal system development,” is relevant to cancer development (Figure 4) [26–28]. Therefore, causative agents of cellular state can be deduced from the subset of differentially coexpressed genes.

### 3.4. Comparisons of Methods and Performance Evaluations

#### 3.4.1. The Results Obtained from the above Analysis Were Compared by a Pairwise Differential Analysis Method

Wu et al. used Cuffdiff to identify the differentially expressed genes (DEGs) of the dataset described above (GSE33782) [14]. In total, 1660, 1528, and 941 genes were extracted as significantly DE between the C-P tissue pairs, the C-N tissue pairs, and the P-N tissue pairs, respectively. Each of these groups contains upregulated and downregulated genes, thus making subsequent functional analysis more complicated. In our approach, genes were classified into 16 categories according to their expression patterns and further stratified based on differences (Figure 2(a)). Finally, the results of the FEA of the two methods were compared (Table S3). According to Wu et al., 31 GO terms in the biological process category were enriched. In total, 17 of 31 GO terms were significantly enriched in our method (FDR ≤ 5.0, Table S3), which were highly relevant to cancer development, such as “collagen metabolic process,” “cell migration,” and “ectoderm development” [29–31]. Little direct evidence was present linking the other categories to cancer; these categories included “heart development,” “regulation of system process,” and “muscle organ development,” which were not significantly enriched in our results (FDR > 5.0, Table S3). Additionally, we discovered some extra categories significantly related to cancer development, such as “blood vessel development,” “collagen metabolic process,” and “cell adhesion” (Figure 4) [32, 33]. The COGO method is based on specifying coexpression patterns to identify function and disease relationships. Therefore, our approach may correctly identify more biological functions than approaches based on pairwise DE methods.

#### 3.4.2. A Comparison with the Direct Clustering Method

A comparative study was performed to evaluate with a direct clustering method using the colon cancer dataset. We first log-transformed (base 2) the gene expression values [34]. A hierarchical cluster analysis with identical settings to the method we developed was applied, and the genes were clustered into 16 categories using the default cluster number formula described above (see Section 2). Our approach displayed a better mining of the coexpression patterns of the transcriptome by reporting a smaller average variable coefficient (CV = 0.24) compared to direct cluster method (CV = 2.10) (Figure S1 and S5).

#### 3.4.3. Comparison with STEM

Simultaneously, we compared our results with the STEM method [35]. The STEM method was developed for short time series microarray datasets and is widely used. The colon dataset was applied under standard procedures of STEM. Notably, genes were also clustered into 16 categories (SC0–SC15, Figure S4), and the average CV of the relative expression of patterns had no significant difference from our method (COGO: 0.24 versus STEM: 0.21) (Figure S5). However, our method provided clearer and more specific coexpression patterns for downstream analysis (Figure S4).

#### 3.4.4. A Time Series Dataset

To further illustrate the performance and the application of our method, a rat pineal gland RNA-Seq dataset with 6 sampling time points was analyzed (GSE46069) [36]. In total, 8,250 genes were obtained after preprocessing, and 27 coexpression patterns were identified by COGO using default settings. A comparative study was provided to compare our method to the direct clustering method. The chart in Figure S6 shows that our results described the data better than the direct clustering method (COGO: CV = 0.27 versus direct clustering: CV = 1.92). One category containing the timekeeping AANAT gene was mainly enriched in the two-function model (Figure S7). One of the functions was related to “cytokine response,” including “response to hormone stimulus” and “response to inorganic substance,” and the other function was related to “neuron function,” including “neuron development” and “axonogenesis.” Both of these functions are associated with the circadian clock, and the findings are consistent with previous studies [37–39].

## 4. Discussion and Conclusions

The transcriptome reveals the status and functional mechanism of the cell as the cell responds to external stimuli. In the presence of various confounders, such as the technical deviation between runs and biological variability, one of the challenges in RNA-Seq data analysis is to extract real biological responses from substantial amounts of transcriptomic expression data. Most of the RNA-Seq data analysis methods have been developed to determine the lists of genes with significant differential expression [40]. In addition, evidence has shown that genes with similar expression patterns are likely to be regulated through similar mechanisms [3]. Alterations in the biological function can be detected by identifying gene expression patterns among a series of RNA-Seq data.

In general, analyses of the transcriptome should be performed on three levels: probe the tendency of macroscopic expression changes, such as in a functional enrichment analysis; analyze captured genes with fluctuations among conditions; and state information based hypotheses and confirm with biological experiments or literature. This research design is a continuously exploring process that cyclically considers the entire dataset to individual members. In this study, all of the detectable genes are stratified into categories according to their expression pattern. A GO enrichment analysis was then performed on each category. We downplayed the importance of DE genes and rediscovered significant gene sets at the integral level. Therefore, the map reflecting biological functional changes is objectively structured on total detectable genes. Genes with different expression patterns exhibit different functional orientations. Therefore, GO terms enriched from categories with large gene-expression differences among conditions may reflect biological dysfunction, and GO terms enriched from categories with little gene-expression differences among conditions may also provide important biological information and may be important for cell survival. Therefore, all of the enriched functional results promote a comprehensive understanding of the molecular mechanisms involved in a specific biological process or disease.

Furthermore, not every category displayed enriched GO terms. Confounding genes may display similar expression patterns and lead to an indeterminate functional orientation or a strong relationship between genes and experimental factors is absent. Our research strategy removes distractions to focus on the notable genes and biological functions. However, meaningful genes can be retrieved through an analysis of significant biological functions or pathways, even in the presence of the unannotated genes.

In this study, we provide an integrated global strategy for coexpression pattern stratification and GO functional analysis for a RNA-Seq data series. We globally clustered genes in RNA-Seq data according to their expression patterns and gene expression differences. The results showed that genes with similar expression patterns clustered into categories in multiple characteristic attribute strategies. This creates opportunities for integrated genomic analyses of unprecedented scope and scale. Global functional analyses can be conducted, and the resulting functional modules provide a diverse repertoire of biological states of different cell types that cannot be captured by analyzing differentially expressed genes alone. Additionally, genes of a specific function can be clustered into categories to explore the expression patterns and regulatory relationships of the functional unit, providing insights into the response of functional mechanisms.

We believe that our method provides a new perspective that downplays the importance of DE genes and rediscovers significant gene sets at an integral level. We provide more useful scenarios for biologists to further explore mechanisms of biological functions and gene regulation.

## Supplementary Material

Figure S1: Compare of the cluster dendrogram of gene expression patterns for COGO method and direct clustering method.
Figure S2: The functional relationship networks of categories and enriched GO terms of molecular function.
Figure S3: The functional relationship networks of categories and enriched GO terms of cellular component.
Figure S4: Comparison of clustering performance for COGO and STEM.
Figure S5: Classification performance comparisons of COGO, STEM and Direct clustering method using the colon cancer dataset.
Figure S6: Performance comparisons of COGO and direct clustering method using the rat pineal gland RNA-seq dataset.
Figure S7: The functional similarity of GO terms of biological process branch from the category C12.
Table S1: The results of co-expression patterns contain original gene expression values, Der, SE and co-expression pattern categories.
Table S2: GO function analysis results of co-expression categories of interest and functional modules recognized by similarity scores.
Table S3: The comparison table of GO enrichment analysis work of pairwise DE method and COGO method.
